# A comparison of team-based learning and lecture-based learning on clinical reasoning and classroom engagement: a cluster randomized controlled trial

**DOI:** 10.1186/s12909-021-02881-8

**Published:** 2021-08-21

**Authors:** Yunefit Ulfa, Yukari Igarashi, Kaori Takahata, Eri Shishido, Shigeko Horiuchi

**Affiliations:** Graduate School of Nursing Science, St. Luke’s International University, Tokyo, Japan

**Keywords:** Active learning, Clinical reasoning, Classroom engagement, Education, Lecture-based learning, Midwifery, Nursing, Cluster randomized controlled trial, Team-based learning

## Abstract

**Background:**

The lecture-based learning (LBL) implemented in most Indonesian nursing/midwifery schools underlies the students’ lack of ability in clinical reasoning. Team-based learning (TBL) was proposed to improve the students’ ability in clinical reasoning as it is applying a course concept of real complex scenarios. In this study, we aimed to assess and compare the effects of TBL and LBL of postpartum hemorrhage topics on the clinical reasoning and classroom engagement of midwifery students in Indonesia.

**Methods:**

We conducted a cluster randomized controlled trial to compare the effects of TBL and LBL. The unit was schools and random allocation was conducted using a simple random sampling method (i.e., coin flipping). There was 1 cluster in the intervention group (n = 62 students) and 1 cluster in the control group (*n* = 53 students). The students in the intervention group participated in a TBL class (90 min) three times, whereas the students in the control group attended an LBL class on postpartum hemorrhage topics. The primary outcome was the *clinical reasoning on postpartum hemorrhage score* measured at pre-test, post-test, and 2 weeks post-test. The secondary outcome was *Classroom Engagement Survey (CES) score* measured after each class finished. We used an unpaired t-test to evaluate the differences between the two groups. The baseline characteristics of the participants were compared using standardized difference.

**Results:**

We evaluated a total of 115 participants. Regarding the baseline characteristics, there was a small difference in the age, Grade Point Average and knowledge at pre-test between the intervention and control groups. The mean clinical reasoning on postpartum hemorrhage scores were significantly higher in the TBL students than in the LBL students at post-test (*p* < .001; Cohen’s d = 1.41) and 2 weeks post-test (*p* < .001; Cohen’s d = 1.50). The CES showed a significantly higher in the intervention group than in the control group.

**Conclusions:**

TBL is an effective learning method for enhancing the clinical reasoning ability of students. This learning method allows for more independent and active learning. Having a strong background knowledge, and discussing cases comprehensively with peers can sharpen the clinical reasoning ability of students.

## Introduction

Postpartum hemorrhage (PPH) is a primary cause of maternal mortality worldwide, primarily in low- and middle-income countries [1]. In Indonesia, the maternal mortality rate in 2015 was 305 out of 100,000 live births [2], 40 % of which was caused by maternal haemorrhage [3]. The two factors that influence PPH severity are the women’s characteristics and deliveries, and medical care [4]. The other main factors that influence the quality of medical care are the medical staff’s knowledge, clinical reasoning, and skills [4–6]. Their lack of ability in preventing and managing hemorrhage often stems from their poor interpretation and implemention of guidelines in handling hemorrhage.

A growing problem in Indonesia is the increasing rate of maternal mortality from PPH despite the increasing number of skilled birth attendants (i.e., doctors, nurses and midwives) [2, 7]. Clinical reasoning plays an important role in nurses’ and midwives’ practice, and this skill depends on their knowledge and experience [8]. However, the absence of tools to assess the clinical reasoning skills of nurses and midwives and the lack of an established teaching approach to develop these skills make assessment by educators difficult. Hence, educators need to develop feasible learning strategies in nursing and midwifery education to produce qualified graduates with good clinical reasoning skills for making logical clinical judgments [9, 10]. Integrating active learning strategies into the nursing and midwifery curriculum is one of the main approaches to enhancing clinical reasoning skills [11]. In Indonesia, however, most forms of nursing and midwifery education still use passive learning strategies, resulting in low-quality midwifery students and a stagnant midwifery practice [12–14].

Team-based learning (TBL) is an active learning method with a sequence structure of activities in and out of the class carried out in small groups. TBL appears to be appropriate in increasing the knowledge of students and in enhancing their clinical reasoning skills as it emphasizes the application of course concepts of real complex scenarios [15, 16]. To our knowledge, studies on the application of TBL to nursing and midwifery education in Indonesia have not yet been conducted. Herein, we implemented TBL on PPH topics in nursing and midwifery education in Indonesia. The present study is a sequel of our study on TBL implementation among Indonesian midwifery students. In our previous study, we focused on students’ knowledge and learning satisfaction, and showed a significantly higher knowledge score and retention in the TBL group than in the lecture-based learning (LBL) group [17]. In the present study, we aimed to assess and compare the effects of TBL and LBL of PPH topics on the clinical reasoning and classroom engagement of midwifery students in Indonesia. The primary outcome was *clinical reasoning on PPH score* and the secondary outcome was *Classroom Engagement Survey (CES) score*.

## Methods

### Design and settings

We conducted a cluster randomized controlled trial. Cluster randomization was adopted to avoid any potential contamination bias at the individual level. The unit was schools and random allocation for the intervention and control groups was conducted using a simple random sampling method (i.e., coin flipping). The allocation was performed before obtaining personal consent. The present study was conducted at two schools of the Midwifery Department of Health Polytechnic Padang, a higher education institution under the Ministry of Health of the Republic of Indonesia. Both schools are under one institution but are located in different cities. One school is located in a sea site and the other in a mountain site. The distance between the schools is approximately 100 kms. We performed this study from September 2019 to November 2019.

### Outcomes

The primary outcome was *clinical reasoning on PPH score* measured at pre-test, post-test, and 2 weeks post-test. The score ranged from 12 to 60. The secondary outcome was *CES score* measured three times after each class session was finished on weeks 1, 2, and 3. The score ranged from 5 to 40. The potential confounders were age and Grade Point Average which were included in the demographic data of the questionnaire.

### Sample size

Although the sample size was determined by the class size, a power calculation indicated that the present sample size was sufficient to assess the effects of the TBL intervention. We calculated the sample size based on the t-test results of our previous pilot study using G power analysis [18]. The effect size was 0.5 at a power of 80 %. The alpha level was set at 0.05, and two independent means in the t-test were used. The estimated sample size was 102. Considering a dropout rate of 10 % based on previous research and the long study duration, we calculated the total sample size as 112 (56 for the intervention group and 56 for the control group).

### Participants

The paricipants were second-year diploma level midwifery students. The inclusion criteria were as follows: (a) graduated from a senior high school (without a nursing background), (b) have no experience of TBL, and (c) completion of the previous academic semester. The exclusion criteria were as follows: (a) graduated from a nursing school, (b) have prior experience of TBL, and (c) noncompletion of the previous academic semester.

### Types of intervention

We chose PPH as the class topic. The content of the PPH material included risk factors, signs, diagnosis, and management/care. The content was designed by the lead researcher (YU) based on the learning objectives and was refined upon consultation with two midwifery experts (KT and SH). One week before the intervention, we provided both groups with a pre-preparation class to set the proper schedule, distribute the syllabus and handout, and explain the TBL procedure to the intervention group.

In the intervention group, we conducted the TBL class in three sessions (one session per week for 90 min). We provided the students a reading assignment about PPH at the pre-preparation class one week before the TBL class. During the TBL class, the lead researcher acted as a facilitator and started the lesson by explaining the learning objective (5 min), followed by the students taking the individual Readiness Assessment Test (iRAT) (10 min) and team Readiness Assessment Test (tRAT) (15 min). The iRAT and tRAT use the same questions consisting of 10 multiple-choice questions without any accompanying notes, books, or other resources. We used the Immediate Feedback Assessment technique form for the tRAT. The students discussed the answers within their teams and then scratched their selected answer on the form. The appearance of a star on a scratched answer indicated a correct answer. If there was no star after scratching, the teams continued to discuss and then selected and scratched off another answer until they obtained the correct answer. At this time, while the students were working on the tRAT, a teaching assistant checked their iRAT answers using a scanning machine and recapitulated which answers were most likely incorrect.

After the tRAT, the teams had the opportunity to submit a written appeal (if needed) (5 min) for incorrect questions or answers with supporting references. The group received additional points if the appeal was accepted, and the facilitator gave a clarification at the next class session. Subsequently, a mini-lecture was given to the students (15 min) regarding the five questions receiving a low score. Finally, an application exercise using vignette questions that applied the topic concepts was distributed and the teams discussed the case (10 min). After each team had discussed the case, inter-team debates were started (25 min). Each team reported their answers and viewpoints to the whole class. In the next class, the same TBL process was used.

In the control group, the PPH topics were delivered using LBL. The lectures were held in three sessions (one session per week for 90 min). On the day of class, the class proceeded as usual. The facilitator (i.e., lead researcher) explained the learning objective (5 min), delivered the content of the material using PowerPoint slides (70 min), and engaged in class discussion (question-and-answer session) (15 min). A question-and-answer session was available during the lecture class, and discussion was allowed if the students wanted to express their opinion or respond to the questions of a peer.

### Data collection and instruments

After obtaining permission to collect data in September 2019, the lead researcher and a research assistant verbally provided details of the study during the class hour, as well as the inclusion and exclusion criteria to second-year midwifery students. They also posted the information on the school’s communication board. The students who agreed to participate in the study signed an informed consent form and returned it on a designated box provided at both schools. We analyzed the clinical reasoning data collected at pre-test, post-test, and 2 weeks post-test. We also conducted CES after completing each class. We distributed a questionnaire which was immediately answered by the students in the same paper. As for clinical reasoning, the students provided a response in the form of an essay. Regarding the CES, the students encircled the correct answer. We provided individual students with their own identification number to blind their personal information in the answer sheet both for the clinical reasoning and the CES.

### Clinical reasoning

The lead researcher used four items [i.e., “How have you interpreted the given information?” (Question no. 1), “How do you link the signs and symptoms of the patient together?” (Question no. 4), “What do you think had happened to the patient?” (Question no. 5), and “What did you aim to do for the patient and why?” (Question no. 6)] of the Clinical Reasoning Evaluation Simulation Tool (CREST) developed by Liaw et al. [19] adjusted to the present study to assess the clinical reasoning skills of the students. This tool had a content validity of 0.93, was obtained from the validations of 15 international experts, had construct and concurrent validity that was supported (*p* < .001), and had a predictive validity that was supported with an existing tool. The Cronbach’s alpha was 0.92 [19]. The lead researcher developed three vignettes on PPH (Case 1: Uterine atony; Case 2: Perineal rupture; Case 3: Endometritis). The vignette scenarios were developed by referring to the learning objectives, readiness assessment test, and national midwife competency test in Indonesia. These vignette scenarios were consulted with four midwifery experts including two faculty members in the setting institutions. The four dimensions of the questionnaire (i.e., data interpretation, signs and symptoms, diagnosis, and treatment) were scored using a five-point Likert scale. Two midwifery faculty experts (HM and YS) marked the student answers using the Likert scale in separate rooms. The results from both midwifery faculty experts were calculated and the mean scores were used. The potential score ranged from 12 to 60. The accomplisment rate was 30.00 (50 % from the highest score). A pilot study was conducted to assess the validity and reliability of the questionnaire and study protocol.

### Classroom engagement survey

CES, which contained eight items, was used to assess student engagement in class [20]. The questionnaire asked about the session that has just finished for a particular day. Items were scored on a five-point Likert scale, and the scores ranged from 5 to 40. A higher score indicated greater engagement, and a score of 24 was considered as a neutral score. The Cronbach’s alpha of CES was 0.881 for undergraduate nursing students [21].

### Data analysis

We used the Statistical Package for the Social Sciences (SPSS) 22.0 (windows) for data analysis. We analyzed demographic data using descriptive statistics. Baseline comparison of the two groups was assessed using standardized difference. We considered a standardized difference > 0.10 as imbalance as to consider adjustment in the analyses. In addition, effect sizes were calculated using Cohen’s d. A larger value of effect size indicates a stronger effect. Effect size was used as an additional control test besides the p-value to interpret data and draw conclusions. The differences in the measurements of clinical reasoning at pre-test, post-test, and 2 weeks post-test measured three times were analyzed using the repeated measure test. The statistical significance was set at *p* < .05 with CI 95 %.

### Ethical consideration

This study was performed in accordance with the provisions of the Declaration of Helsinki. This study was reviewed and approved by the Institutional Review Board of the Ethics Committee of St. Luke’s International University, Japan (No. 19-A055). The study was registered in University Hospital Medical Information Network (UMIN) (UMIN000038062). All participants provided informed consent prior to participation in the study.

## Results

**Fig. 1:**
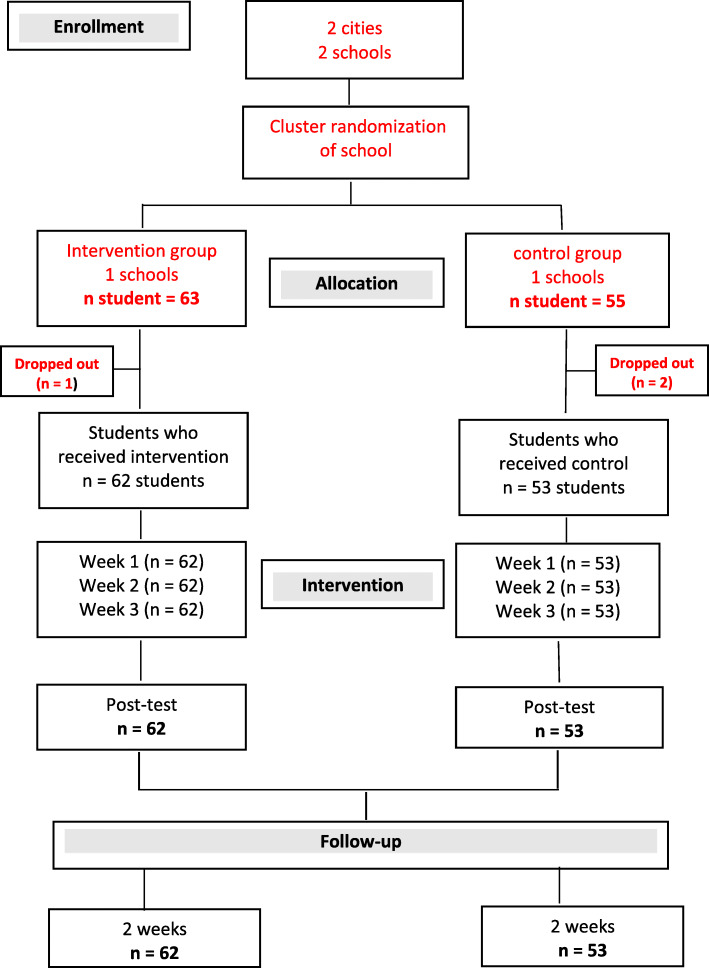
Participant flow diagram

Of the 118 midwifery students who were assessed and met the eligibility criteria, 115 (97.5 %) participated in the study. Of these 115 students, 62 students from the mountain site setting were enrolled in the intervention group and participated in the TBL classes, and 53 students from the sea site setting were enrolled in the control group and received the LBL classes. The participant flow diagram is shown in Fig. 1.

### Characteristic of participants

The baseline characteristics of the participants were compared between the control group and the intervention group (Table 1) using standardized difference. There was a small difference in the age. Although the standardized differences in the Grade Point Average (GPA) and knowledge at pre-test between the intervention group and the control group were > 0.10, the values did not deviate greatly from 0.10.

**Table 1:** Comparison of the baseline characteristics of the participants in the intervention and control groups

	Intervention (*n* = 62)	Control *(n = 53)*	Standardized difference
**Age (years) (*SD*)**	19.19 (0.54)	19.15 (0.50)	0.07
**Educational level**			
Senior high school (%)	62 (100 %)	53 (100 %)	0
**Future clinical midwife profession**			
Work as practitioner (%)	57 (91.9 %)	41 (77.4 %)	0.41 0.30 0.26
Work as an academic (%)	3 (4.8 %)	7 (13.2 %)	
Others (%)	2 (3.2 %)	5 (9.4 %)	
**Grade Point Average (GPA)**			
With honors [3.51-4.00] (%)	31 (50 %)	30 (56.6 %)	0.13 0.130
Very Satisfactory [2.76–3.50] (%)	31(50 %)	23 (43.4 %)	
Satisfactory [2.00-2.75] (%)	0 (0 %)	0 (0 %)	
**Knowledge (Pre-test; Range 1-100) (Mean; SD)**	45.94 (13.29)	43.29 (18.89)	0.16

### Primary outcome: Clinical reasoning on postpartum hemorrhage score

The comparison of the mean *clinical reasoning on PPH scores* at pre-test, post-test, and 2 weeks post-test is shown in Table 2. There was no significant difference in the mean clinical scores at pre-test between the intervention group and the control group (*t* = 1.86; *p* = .65, Cohen’s d = 0.35). After the interventions, the mean *clinical reasoning on PPH scores* of the TBL group were significantly higher than those of the control group at post-test (*t* = 7.52; *p* < .001, Cohen’s d = 1.41) and 2 weeks post-test (*t* = 8.0; *p* < .001, Cohen’s d = 1.50) at all subscales.

We used the knowledge score data from our previous study [17] for secondary analysis to assess the correlation of knowledge with clinical reasoning on PPH. The results showed that the knowledge and clinical reasoning on PPH scores showed a significant correlation at post-test (r = .488, *p* = .000) and at 2 weeks post-test (r = .522, *p* = .000).

**Table 2:** Comparison of the mean clinical reasoning scores between the intervention group and the control group

	Intervention(*n* = 62)	Control(*n* = 53)	*T*^*a*^	*p*-value	Effect Size (Cohen’s d)
Pre-test (range 1–60)	18.31 (SD = 5.43)	16.58 (SD = 4.30)	1.86	0.65	0.35
Data interpretation (1–5)	1.55 (SD = 0.88)	1.34 (SD = 0.62)	1.49	0.140	
Signs and symptoms (1–5)	1.79 (SD = 0.55)	1.62 (SD = 0.49)	1.72	0.088	
Diagnosis (1–5)	1.55 ((SD = 0.56)	1.38 (SD = 0.56)	1.62	0.107	
Treatment (1–5)	1.47 (SD = 0.62)	1.25 (SD = 0.48)	2.17	0.032	
Post-test (range 1–60)	38.0 (SD = 7.36)	28.55 (SD = 5.89)	7.52	< 0.001	1.41
Data interpretation (1–5)	3.71 (SD = 1.27)	2.87 (SD = 1.00)	3.89	< 0.001	
Signs and symptoms (1–5)	3.05 (SD = 0.66)	2.34 (SD = 0.48)	6.47	< 0.001	
Diagnosis (1–5)	3.10 (SD = 0.56)	2.34 (SD = 0.65)	6.62	< 0.001	
Treatment (1–5)	3.10 (SD = 0.62)	2.15 (SD = 0.79)	7.03	< 0.001	
2 weeks post-test(range 1–60)	34.0 (SD = 7.32)	23.81 (SD = 6.16)	8.00	< 0.001	1.50
Data interpretation (1–5)	3.82 (SD = 1.25)	2.53 (SD = 0.99)	6.08	< 0.001	
Signs and symptoms (1–5)	2.66 (SD = 0.75)	2.08 (SD = 0.47)	5.10	< 0.001	
Diagnosis (1–5)	2.66 (SD = 0.63)	1.87 (SD = 0.65)	6.65	< 0.001	
Treatment (1–5)	2.44 (SD = 0.78)	1.53 (SD = 0.58)	7.15	< 0.001	

^a^ Unpaired t-test

**Fig. 2:**
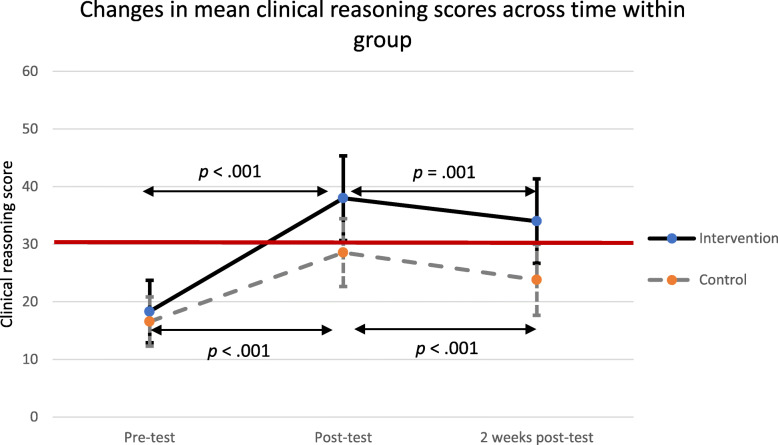
Changes in the mean clinical reasoning scores across time in the intervention group and control group. The error bars indicate the standard deviation. The red line indicates the accomplishment rate (30.0)

Regarding the changes in the mean clinical reasoning on PPH scores within group across time (Fig. 2), there were significant increases in the mean clinical reasoning on PPH scores from pre-test to post-test (*p* < .001) in the intervention and control groups, but slight decreases from post-test to 2 weeks post-test. The accomplishment rate for clinical reasoning on PPH was 30.0. As shown in Fig. 2, the intervention group showed a mean clinical reasoning on PPH score that was higher than the accomplishment rate at post-test and 2 weeks post-test compared with the control group which showed a mean clinical reasoning on PPH score below 30.0 at post-test and 2 weeks post-test. Overall, although the intervention group showed a slight decrease in the mean clinical reasoning on PPH score at 2 weeks post-test, the intervention group still maintained a knowledge score above the accomplishment rate

### Secondary outcome: Classroom engagement survey score

There was a significant difference in the Total CES scores among the CES 1, CES 2, and CES 3 between the intervention group and the control group (Table 3). The intervention group showed significant differences in classroom engagement scores compared with the control group.

**Fig. 3:**
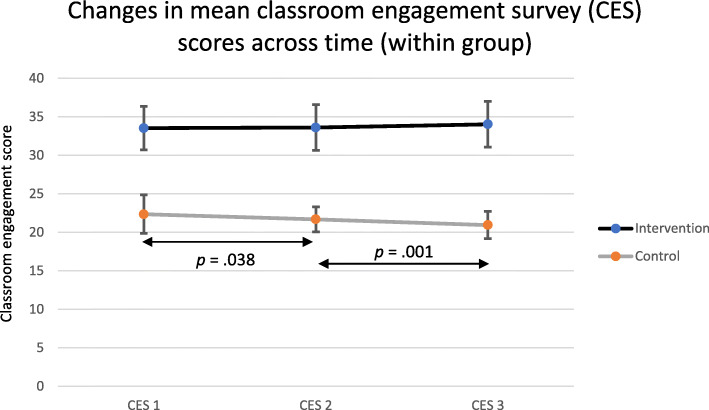
Changes in mean classroom engagement survey scores across time in the intervention group and control group. The error bars indicate the standard deviation

As shown in Fig. 3, there were no significant differences in the mean CES scores from sessions 1 to 2 and sessions 2 to 3 in the intervention group (*p* > .05). There was a significant decrease in the mean CES scores from sessions 1 to 2 (*p* = .038), and from sessions 2 to 3 (*p* = .001) in the control group.

**Table 3:** Comparison of classroom engagement survey items between the intervention and control groups

	Classroom engagement survey (CES)Range: 1–40	CES 1 (Week 1)				CES 2 (Week 2)				CES 3 (Week 3)			
		Intervention	Control	*T*^b^	*p-value*	Intervention	Control	*T*^b^	*p-value*	Intervention	Control	*T*^b^	*p-value*
Q1	Most students were actively involved	4.08 (SD = 0.75)	2.02 (SD = 0.54)	17.07	< 0.001	4.15 (SD = 0.70)	2.11 (SD = 0.58)	17.09	< 0.001	4.27 (SD = 0.63)	1.96 (SD = 0.59)	20.33	< 0.001
Q2	I had fun in class today.	4.61 (SD = 0.55)	3.32 (SD = 0.51)	12.93	< 0.001	4.50 (SD = 0.54)	3.34 (SD = 0.48)	12.27	< 0.001	4.50 (SD = 0.54)	3.25 (SD = 0.52)	12.78	< 0.001
Q3	I contributed meaningfully to class discussions.	3.76 (SD = 0.76)	2.25 (SD = 0.65)	11.51	< 0.001	3.94 (SD = 0.65)	1.98 (SD = 0.42)	19.48	< 0.001	3.82 (SD = 0.64)	1.91 (SD = 0.40)	19.45	< 0.001
Q4	Most students were not paying attention ^a^	4.02 (SD = 0.50)	3.00 (SD = 0.62)	9.76	< 0.001	3.87 (SD = 0.64)	2.75 (SD = 0.52)	10.19	< 0.001	4.08 (SD = 0.61)	2.58 (SD = 0.63)	12.89	< 0.001
Q5	I paid attention most of the time	4.19 (SD = 0.70)	2.89 (SD = 0.47)	11.95	< 0.001	4.11 (SD = 0.68)	2.81 (SD = 0.62)	10.64	< 0.001	4.11 (SD = 0.66)	2.64 (SD = 0.59)	12.55	< 0.001
Q6	I did not enjoy class today^a^	4.39 (SD = 0.61)	3.66 (SD = 0.62)	6.33	< 0.001	4.45 (SD = 0.59)	3.60 (SD = 0.63)	7.43	< 0.001	4.50 (SD = 0.57)	3.60 (SD = 0.69)	7.66	< 0.001
Q7	I participated in the class most of the time.	3.90 (SD = 0.67)	2.11 (SD = 0.54)	15.82	< 0.001	3.89 (SD = 0.66)	2.02 (SD = 0.31)	19.99	< 0.001	4.05 (SD = 0.66)	1.98 (SD = 0.37)	21.06	< 0.001
Q8	I would like more class sessions to be like this one.	4.58 (SD = 0.64)	3.09 (SD = 0.63)	12.5	< 0.001	4.71 (SD = 0.55)	3.06 (SD = 0.57)	15.74	< 0.001	4.69 (SD = 0.53)	3.02 (SD = 0.57)	16.28	< 0.001
	Total	33.53 (2.83)	22.34 (2.50)	22.27	< 0.001	33.61 (2.96)	21.68 (1.62)	26.19	< 0.001	34.03 (2.98)	20.94 (1.77)	28.01	< 0.001

*Note.* Likert scale (1, strongly disagree; 2, disagree; 3, neither agree nor disagree; 4, agree; 5, strongly agree). Neutral = 24

^a^*means the score of reverse items has been change.*

^b^*Unpaired t-test*

## Discussion

The aim of this study was to assess and compare the effects of TBL and LBL of PPH topics on the clinical reasoning and classroom engagement of midwifery students. The primary outcome was *clinical reasoning on PPH score* and the secondary outcome was *CES score.*

### Clinical reasoning on PPH between TBL and LBL

The present results showed a significant difference in the student clinical reasoning on PPH between the intervention and control groups just after the intervention. The intervention group had a higher clinical reasoning on PPH score than the control group. This result is supported by the results of a previous study [22] on nursing students which reported that TBL strategies improve clinical reasoning. The study of Okubo et al. [23] involving fourth-year medical students showed that TBL is helpful in improving the clinical reasoning ability of students with Problem-Based Learning experiences but limited clinical exposure.

Another study on undergraduate medical students described that students in the TBL class have a better performance in clinical reasoning using key feature problem examination (KFPE) than students in interactive seminars [16]. Similarly, Jost et al. [24] conducted a study involving 26 fourth-year and fifth-year medical students in Germany. They found that the TBL group performed significantly better than the non-TBL group in KFPE. Moreover, Tan et al. performed a modified crossover study involving 179 third-year and fifth-year undergraduate students from the School of Medicine in Singapore [25]. They found that TBL was slightly better than interactive lectures in enhancing student clinical reasoning for a particular subject.

Compared with a previous study that assessed clinical reasoning using KFPE, the clinical reasoning in the present study was measured using the CREST which comprehensively assesses the students’ ability in solving cases and the students’ analytical thinking in linking signs and symptoms to appropriate diagnoses and actions according to the scenario provided. A systematically written essay as answer is required. A student’s analytical thinking is assessed very well as close as possible to the real situation.

We previously showed that students in the intervention group (TBL) had a higher knowledge score than students in the control group (LBL) [17]. This result also indicates that knowledge has a significant correlation with clinical reasoning. According to Benner et al. [26], clinical reasoning is the ability to integrate knowledge and critical thinking. In the TBL process, the application exercise applies the topic concepts, stimulating students to use their knowledge and to think critically. This enhances their clinical reasoning ability. The facilitator gives the vignette scenario of a common case on the practice area. The students then discuss within their group and participate in inter-team debates. Application exercises are more prioritized than simply memorizing knowledge, driving students to think more critically during problem solving [27]. In addition, Prior out-class reading, iRAT, and tRAT gently help students sharpen their ability to decipher the clue and interpret the case during clinical reasoning [23].

Figure 2 showed that although the intervention group had a slighly decreased score, the overall score was above the accomplishment rate. This indicates that the intervention group still maintained good clinical reasoning compared with the control group. Handling obstetric emergencies is a subtantial work for nurse-midwives in the community. In the present study, the questions on risk factors, causes of PPH, diagnosis, and care/treatment required knowledge and clinical reasoning. Therefore, students must think critically to integrate their acquired knowledge and make appropriate clinical reasoning. A systematic review covering 29 countries found that the identification and management of PPH were poor among healthcare providers in these countries [28]. Therefore, the application of TBL in the teaching-learning process is assumed to increase the ability of nurse-midwives to more appropriately evaluate and manage PPH.

Brewer [22] indicated the need to change the pedagogical strategies in nursing education towards more interactive and student-centered learning strategies. An example is the use of TBL which enhances the students’ clinical reasoning as TBL overcomes any barrier between the course substance and the application from the classroom to the clinical setting. Recent studies have also developed online TBL in medical education to address pedagogical needs brought about by the pandemic situation. In their studies, Jackson et al. [29] and Gaber et al. [30] reported that TBL received the highest student satisfaction rate, and that TBL promoted critical thinking and self-directed learning even in virtual learning.

### Classroom engagement survey

We found a significant difference in student classroom engagement between the intervention and control groups. The intervention group showed a higher classroom engagement than the control group. This finding was similar to that of a previous study which found that TBL was more engaging than traditional teaching in adult health nursing, maternal-child nursing, community health nursing, and medical-surgical nursing [31]. Moreover, several studies in nurse education have shown that TBL increased student engagement [32–34]. In TBL, the students are required to share their knowledge and ideas, then discuss the content material with their peers. In tRAT activities, for instance, students have to discuss the questions to get one correct answer with their team, as well as in the appeal process and application exercise. Thus, the TBL activities were the underlying reasons why the students were more engaging in the classes.

Regarding the CES, the item “*I had fun in class today*” had a higher mean score in the TBL group than in the control group. Previous studies have found that students enjoyed studying in teams because they can articulate their ideas or ask questions among their peers without anxiety [35, 36]. The students in the TBL group strongly agreed to have more TBL classes than the students in the control group who agreed to have more lecture classes. This means that students who have experienced lectures and TBL classes prefer to have more TBL sessions in other courses. However, because of the absence of the TBL experience in the control group, the students still learned through traditional lecture.

The students in the TBL group highly contributed to the class discussions compared with the students in the traditional lecture group. Most of the students in the control group declined to give a contribution to the class discussion. The reason for the enhanced contribution by the students in the TBL group is that most of the time, they were asked to have a discussion, in contrast to the traditional lectures wherein the students only learned passively. Therefore, the TBL activities showed how TBL can promote classroom engagement.

### Strenghts and limitations

Regarding the strengths of this study, the participants were from two different schools under the same institution with the same system and regulations. Thus, the characteristics of the intervention and control groups are almost the same. Moreover, the distance between the two schools was sufficiently far to minimize contamination between the two groups. In addition, the participants used an ID number on their answer sheet. Therefore, examiners who assessed the students’ clinical reasoning were completely blinded.

The limitation of this study was the short session of TBL which was not an optimal duration of exposure for the most effective delivery of TBL. In the near future, we need to apply TBL for at least one semester to realize the maximal outcome of this learning method. Moreover, the teacher’s role driven by the researchers may affect the intervention accidentally.

Furthermore, extending the clinical reasoning duration up to 6 or 12 weeks instead of up to the post-test period can be considered to facilitate the assessment of retention of the content to enable the students to deepen and maintain their understanding. Oral assessment or a clinical practice test can be used as a companion of the writing test to enable a more accurate assessment of the students’ clinical reasoning ability.

## Conclusions

This study assessed and compared the effects of TBL and LBL of PPH topics on the clinical reasoning and classroom engagement of midwifery students in Indonesia using a cluster randomized controlled trial. *Clinical reasoning on PPH score* and *CES score* served as primary and secondary outcomes. The study showed that the midwifery students in the TBL group had a higher *clinical reasoning on PPH score* than the midwifery students in the LBL group. The midwifery students in the TBL group could also maintain their clinical reasoning after two weeks from the intervention. The *clinical reasoning on PPH* showed a positive correlation with knowledge of PPH. The midwifery students in the TBL group also had a higher *CES score* than the midwifery students in the LBL group. These findings indicate that TBL is an active learning method that can enhance midwifery students’ clinical reasoning on PPH and classroom engagement compared with the traditional LBL.

For subsequent studies, the optimal duration of TBL exposure should be explored to determine the most effective delivery of TBL and realize its maximal outcome. Extension of the clinical reasoning duration should also be investigated to facilitate assessment of content retention and understanding of students.

## Data Availability

The datasets used and/or analyzed during the current study are available from the corresponding author upon reasonable request.

## Abbreviations

CES: Classroom Engagement Survey
CREST: Clinical Reasoning Evaluation Simulation Tool
GPA: Grade Point Average
iRAT: Individual Readiness Assurance Test
KFPE: Key feature Problem Examination
LBL: Lecture-based learning
PPH: Postpartum Hemorrhage
SPSS: Statistical Package for the Social Sciences
TBL: Team-Based Learning
tRAT: Team Readiness Assurance Test
UMIN: University Hospital Medical Information Network
